# Supplementary material for: Are carbon emissions trading and green financial instruments synergistic? -Comprehensive quantitative research based on content analysis
Source: PLoS One. 2024 Mar 7;19(3):e0298601. doi: 10.1371/journal.pone.0298601 (PMC10919601; doi:10.1371/journal.pone.0298601)
Supplement: S1 Data — (ZIP) [file pone.0298601.s001.zip › Data/地级市Z_F工作报告.zip!/╘╞─╧/▒ú╔╜╩╨/└┤╫╘╣1⁄2╓┌║┼í╛┬φ┐╦╩2╛▌═°í┐_2017.docx]

各位代表：

现在，我代表市人民政府，向大会报告工作，请予审议，并请市政协委员和列席人员提出意见。

一、过去五年工作回顾

过去五年，在省委、省政府和市委的坚强领导下，市人民政府团结和依靠全市各族干部群众，主动服务和融入国家“一带一路” 战略，积极适应经济发展新常态，有效应对经济下行压力，全市经济社会发展成效显著。

五年来，综合经济实力进一步提升。我们始终把发展作为第一要务，主要经济指标增速连续多年位居全省前列。生产总值从323.2亿元增加到613.4亿元，年均增长12.4%；人均生产总值从12847元增加到23692元，年均增长11.8%。财政总收入从47.4亿元增加到86.5亿元，一般公共预算收入从28.2亿元增加到57.5亿元，年均分别增长12.8%和15.3%；一般公共预算支出从106.4亿元增加到212.7亿元，年均增长14.9%。农村常住居民人均可支配收入从4439元增加到9426元，年均增长14.8%；城镇常住居民人均可支配收入从16228元增加到27801元，年均增长11.4%。社会消费品零售总额从101亿元增加到200亿元，年均增长13.9%。金融机构各项存款余额从415亿元增加到986亿元，各项贷款余额从269亿元增加到599亿元，年均分别增长18.8%和17.3%。引进市外到位资金642亿元，年均增长39.2%。非公经济增加值从128亿元增加到307亿元，占生产总值比重达50.1%。产业结构由30.4：33.1：36.5 优化为24.7：34.9：40.4。

五年来，发展条件进一步改善。我们坚持把打基础作为首要任务，进一步建立和完善项目投资工作机制，加强重大项目建设。累计完成固定资产投资2063亿元，年均增长32.6%，是“十一五”的2.9倍。以综合交通、城镇为重点的基础设施建设取得重大突破。保腾高速、龙瑞高速及8条二级公路建成通车，新增公路通车里程1241公里，乡镇全部通沥青或水泥路，建制村公路通畅率达97.8%，昌宁、施甸、龙陵率先实现建制村全部通畅，位居全省前列；大保铁路建设加快，腾冲机场改扩建工程稳步推进；槟榔江三岔河大（二）型水库等一批水利设施建成，水库库容达9.69亿立方米，解决71万农村人口饮水安全问题；建成220千伏输变电站4座、110千伏35座，新增电力装机容量72万千瓦，达212万千瓦，中缅天然气管网保山支线建设进展顺利，能源保障能力持续提升；信息惠民国家试点城市、信息产业园建设扎实推进，五大数据库基本建成，4G网络、光纤宽带覆盖范围逐步扩大。创新型保山建设稳步推进，国家级高新技术企业达41户，建立院士专家工作站10个，腾冲经开区、保山工贸园区获批省级高新技术产业开发区。城乡面貌变化喜人，建成地下综合管廊48.3公里，位居全省前列，城市路网、“三校一院”等基础设施建设同步推进。新增城市建成区面积21.55平方公里，达81.55平方公里，城镇化率达43.6%，提高11.9个百分点。

五年来，民生保障水平进一步提高。累计支出民生资金660.7亿元，占财政支出的76.7%。脱贫攻坚扎实推进，42.7万贫困人口实现脱贫。新增就业岗位12万个，城镇失业率控制在4%以内。各级各类教育质量不断提高。城乡居民基本医疗、养老保险制度实现全覆盖，城乡低保补助标准提高1倍。建设保障性住房4.5万套，累计分配4万套。实施棚户区改造2.76万套（户）。成功创建国家公共文化服务体系示范区，被评为全国文化体制改革先进地区，杨善洲精神教育基地、滇西抗战纪念馆等一批文化设施建成使用。体育设施建设加快，建成2个县级全民健身中心、3个健身公园。生态市创建稳步推进，森林覆盖率提高3.1个百分点，达65%，城乡人居环境明显改善。

五年来，民主法治建设进一步加强。认真执行市人大及其常委会的各项决议决定，自觉接受市人大及其常委会的法律监督、工作监督和市政协的民主监督，5年累计办理建议413件、提案877件，办复质量和时效不断提高。全面推进依法治市，普法工作成效明显。龙陵黄龙玉资源管理条例颁发实施，昌宁田园城市保护等地方立法积极推进。法治、责任、阳光、效能政府建设卓有成效。民族团结进步示范区创建深入推进，少数民族地区经济社会发展加快，民族团结、宗教和谐。国防建设不断加强，军警民团结的良好局面更加巩固。信访和群众工作得到加强，食品药品安全和安全生产形势持续好转。“保山经验”入选“全国社会治理创新案例”，社会治安立体化防控水平不断提升，群众安全感满意度居全省前列。与此同时，人防、档案、地方志、红十字、老龄、妇女儿童、残疾人等事业健康发展。

各位代表，第三届市人民政府各项目标任务已经顺利完成。本届政府的五年是保山发展最快的时期之一，为全面建成小康社会奠定了坚实基础。

刚刚过去的2016年，我们全力以赴稳增长、促改革、调结构、惠民生、防风险，经济社会发展取得了新的成绩。受国内经济下行等因素影响，除进出口总额、城乡居民收入、银行贷款余额未能完成预期目标外，市三届人大五次会议确定的主要目标任务均已较好完成。

（一）强化政策落实，全力以赴稳增长。全面贯彻国家、省稳增长总体部署，及时出台一系列政策措施，突出在产业培育、资金支持、平台搭建、降低电价、用地保障等方面发力，强化督查考评和激励问责，主要经济指标实现两位数增长，增幅居全省前列。全市实现生产总值613.4亿元，增长11.1%，增幅居全省第一。其中，第一产业增加值151.3亿元，增长6%；第二产业增加值214亿元，增长14.7%；第三产业增加值248亿元，增长11.2%。一般公共预算收入57.5亿元、增长10.1%，支出212.7亿元、增长10.7%。

（二）强化产业培育，力促转型升级。着力培育6大产业，重点产业导向作用初步显现。特色农业发展加快。全力打造施甸万家欢、龙陵禇橙等10个万亩规模农业示范区。粮食总产量143.9万吨，实现13年连增。肉类总产量45.9万吨、畜牧业产值102.4亿元。新增专业合作社315个、省级龙头企业10户、市级龙头企业15户。工业经济健康发展。实行领导挂钩联系服务重点企业、重点项目制度，强化企业生产经营要素保障，实施“一企一策”帮扶。园区建设创新提速，与县市区共建“园中园”，引进企业20户，有力推动了产业集群集约发展。新增规模企业39户，规模以上工业增加值124.7亿元，增长12.2%。建筑业不断壮大，实现增加值69.3亿元，增长22.4%。保山硅材料基地列入国家火炬特色产业基地，新增国家级高新技术企业11户，新建院士专家工作站4个。第三产业发展趋好。现代金融、房地产、物流、电子商务等服务业发展加快，社会消费品零售总额增长12.2%。全域旅游10项重点工作全面启动，火山热海成为滇西第一个5A级旅游景区，高黎贡山国家公园等重点项目积极推进，接待旅游者1833万人次，增长43%。

（三）强化基础设施建设，突出投资关键作用。以综合交通、棚户区改造、市政设施、易地搬迁等为重点，加快推进“五网”等基础设施建设。龙江特大桥建成通车，大瑞铁路澜沧江特大桥钢管拱合龙，腾陇、腾猴、昌保、保施、东绕城高速开工建设，保泸高速、腾冲机场改扩建等工程进展顺利。成功申报全国2016年地下综合管廊试点城市，保山国家级天然气利用试验示范区建设规划和方案获国家批复，腾冲市、板桥镇成为国家新型城镇化综合试点，3个万亩生态廊道工程全面启动，青华海列为国家湿地公园试点，有轨电车项目前期工作扎实推进。新增电力装机容量18.2万千瓦。新开工昌宁立新等8座中小型水库，完成6.2万农村人口饮水安全巩固提升任务。完成规模以上固定资产投资667.3亿元，增长33%，增速居全省第二。

（四）强化改革开放，切实增强发展活力。财税体制改革力度加大，营改增顺利推进，政府债务管理进一步加强。PPP项目、专项建设基金和专项债券争取、“51+49”“10+3”等投融资创新取得成效，设立政府引导性产业基金30亿元、扶贫基金100亿元，争取国家专项建设基金50.4亿元。中心城市地下综合管廊、东绕城高速、昌保高速、6座重点水源工程采用PPP模式建设。出台了 “1+5”的总体意见和专项实施意见，供给侧结构性改革有力推进，降低实体企业生产经营成本16.5亿元。全国质量强市示范城市创建通过省级验收，获“全国质量魅力城市”称号。国资国企、商事制度、农村信用社、教育、卫生、社保、殡葬等领域改革稳步推进。土地承包经营权确权登记颁证等农村综合改革有序开展，流转土地30多万亩。农村公路养护模式得到部、省肯定，并在全省推广，昌宁荣获云南省四好农村公路创建示范县。密班公路建设扎实推进，完成进出口总额2.66亿美元，增长10.1%。

（五）强化民生事业，促进社会和谐。8件惠民实事全部完成。城乡居民人均可支配收入分别增长8.4%、10%。消除农村集体经济“空壳村”73个，在全省率先实现全面消除目标。脱贫攻坚有力推进，争取扶贫资金136.9亿元，完成5个整乡推进、65个行政村整村推进、22个自然村推进项目，实施农村危房改造2.45万户，启动建设易地扶贫搬迁三年行动计划集中安置点200个，4.1万名干部职工开展“挂包帮”“转走访”，9个建档立卡贫困乡出列、80个贫困村退出、6.68万人脱贫。开发就业岗位2.94万个，城镇新增就业2.53万人，城镇登记失业率3.43%。企业退休人员养老金人均增资160元。特困人员救助供养制度进一步健全，城乡低保补助水平分别提高8%和12%。改造义务教育薄弱学校校舍10万平方米，基本完成教学仪器、图书、多媒体设备等配置任务。公办幼儿园和普惠性民办幼儿园在园幼儿占比达93.3%。保一中、中医专、中专学校顺利搬迁，保一中新校区初中部宿舍楼启动建设，滇西应用技术大学珠宝学院实现招生，保山技工学校升级为技师学院。公共卫生服务均等化水平稳步提升，新农合参合率达99%。文化惠民措施有效落实，群众性文体活动广泛开展，成功举办首届国际观鸟节、市第四届运动会、腾冲国际半程马拉松赛，云南保山永子棋队在围甲比赛中取得骄人战绩。深入开展平安保山、法治保山建设，健全“三个一”立体化治安防控体系，严防严打各类违法犯罪；落实信访“三个亲自”，切实解决群众合理诉求；“995”综合为民服务平台进一步扩展、“6995”不断巩固，荣获“全国创新社会治理优秀城市”称号；加大安全隐患排查治理力度，安全生产、食药品安全得到加强，社会稳定和谐。

（六）强化政府自身建设，推进政府职能转变。深入开展“两学一做”学习教育，作风建设常态化机制初步形成，责任落实、奖惩考核、督促检查明显强化，执行力、创新力有效提升。强力推进“放管服”改革，向社会公布市级政府部门权责清单，取消行政审批20项，下放9项，非行政许可审批全部取消。事业单位分类改革、公务用车制度改革稳步推进。积极推动电子政务应用，“双随机一公开”监管方式全面推开，行政监察和审计监督进一步加强，工作效率明显提高。

各位代表，同志们！过去五年所取得的成绩来之不易。在此，我代表市人民政府，向全市各族人民，向各位代表、各位委员，向各民主党派、工商联、无党派人士、各人民团体，向驻保人民解放军、武警官兵和中央、省属各部门各单位，向离退休老领导、老同志以及所有关心支持帮助保山建设发展的社会各界人士，致以崇高的敬意和衷心的感谢！

过去五年，我们所积累的经验弥足珍贵。五年来，我们始终坚持解放思想不动摇，着力破除思想观念、体制机制障碍，以改革创新求突破，不断增强发展的动力和活力；始终坚持跨越发展不停步，好中求快，奋力赶超，推动保山步入发展的快车道；始终坚持扭住重点不松劲，牢牢抓住投资拉动、产业培育两个关键，努力破解发展难题；始终坚持民生为本不偏移，以保障和改善民生为出发点和落脚点，着力提高人民群众生活水平；始终坚持从严治政不懈怠，积极推进政府职能和作风转变，不断提升公信力和执行力，为经济社会平稳较快发展提供有力保障。

在充分肯定成绩的同时，我们也清醒地看到，实现保山跨越发展还存在许多困难和问题，主要表现在：一是基础设施和产业短板突出，跨越发展基础不牢；二是体制机制障碍较多，沿边开放优势尚未形成，跨越发展活力仍未充分释放；三是城乡区域发展不平衡、不协调，脱贫攻坚面广量大，跨越发展任务艰巨；四是思想解放不够，创新能力薄弱，高端人才、产业工人和农村实用人才短缺，跨越发展人才和科技支撑明显不足；五是政府自身建设有待加强，干部的工作能力、工作作风和服务水平与跨越发展要求还有差距。我们将正视这些困难和问题，认真研究解决，努力把各项工作做得更好。

二、今后五年的工作

纵观全局，保山发展机遇十分难得；环顾周边，逆水行舟，不进则退，慢进也是退；审视自身，发展条件正在发生深刻变化，保山追赶跨越正处在关键时期。我们必须解放思想、改革创新，抢抓机遇、乘势而上，凝心聚力、赶超跨越。

今后五年，我们要深入贯彻习近平总书记系列重要讲话精神和治国理政新理念新思想新战略，积极适应把握引领经济发展新常态，统筹推进“五位一体”总体布局，协调推进“四个全面”战略布局，认真贯彻落实好省委省政府和市委的指示要求，围绕建设“六个保山”总体目标，坚持实际论、方法论、创新论、跨越论和比较论，全力实施六大工程，做大做强六大产业，推进农业规模化、工业聚集化、城市生态化、旅游品牌化、棚改货币化和高速公路建设高效化，着力打造全省经济发展新的增长极，争当全省产业转型升级和生态文明建设排头兵，积极面向南亚东南亚开放，开创边疆地区跨越发展新局面。

今后五年的目标建议为：生产总值年均增长11%以上，突破千亿元；人均生产总值年均增长10%以上，达到全省平均水平；三次产业结构调整为20: 40 :40；民营经济增加值占GDP的55%以上；一般公共预算收入年均增长10%以上；固定资产投资年均增长20%以上；社会消费品零售总额年均增长12%以上；价格总水平保持基本稳定；城乡常住居民人均可支配收入年均增长10%和12%以上；进出口总额年均增长20%以上；金融机构存贷款余额分别增长13%和15%以上；城镇化率达50%以上；教育、科技、卫生、文化等基本公共服务能力进一步增强，社会保障体系更加健全。

（一）强力推进投资增长，加强基础设施建设。五年累计完成投资6000亿元以上，努力补齐基础设施短板。加快建设以航空为先导，高速公路、铁路为重点，水运为补充的立体综合交通网络。建成保泸、昌保、保施等7条高速公路和3000公里乡村公路，实现县县通高速路、80%以上的乡镇通二级路、村村通硬化路。建成大保铁路，推进保瑞铁路，推动保山至猴桥、六库铁路建设。建成保山通用航空基地，有序推动昌宁、腾冲、施甸、龙陵通用机场建设。加快在建水电站和骨干电网建设，着力推进输变电工程，积极参与境外水电开发。推进国家级天然气利用试验示范区建设。深入实施“兴水强保”战略，建设一批重点水源水利项目，实施农村饮水安全巩固提升工程。持续抓好棚户区改造，每年完成投资100亿元以上。积极融入全省“云上云”行动计划，加快创建信息惠民国家试点城市，推进智慧城市建设，每个县市区、园区至少建成一个智慧城市试点片区。

（二）强力推进改革开放，创新体制机制。以满足需求为导向、提高质量为核心，深入推进供给侧结构性改革，切实抓好经济体制、行政体制、农业农村、社会事业、社会保障等重点领域改革，持续增强跨越发展的动力和活力。创新投融资机制，实施政府引导性基金、“51+49”“10+3”等合作模式，加强融资平台建设管理，争取扩大财政支持和信贷规模，大力推行PPP模式和政府购买服务，有效激活社会资本，助推民营经济发展。创新园区管理机制，建立重点产业聚集、干部人事分类管理、薪酬激励分配和财政资金扶持、项目集中连片审批等机制，推动园区率先发展。创新城市管理机制，加快推进城市管理数字化，构建城市管理综合指挥和执法体系，完善网格化管理机制，加大市政基础设施管护市场化改革力度，全面提升城市管理服务水平。创新农村土地流转机制，有序引导农村土地向新型经营主体流转，健全利益联结和风险防范机制，促进农业规模化发展和农民增收。创新开放发展机制，加强交通、网络、信息互联互通，提高通关便利化水平，加快转口加工、电子商务、跨境旅游、现代物流等外向型产业发展，推动腾冲边合区、曼德勒保山缪达工业园和产品展示中心、密支那工业园区及沿边金融等平台建设，建立完善与缅、印、孟等交流合作机制，加强农业、经贸、人文等交流合作，轮流举办保山—密支那边境交易会，着力构建丝路永昌开放体系。

（三）强力推进园区建设，着力培育六大产业。优化产业空间布局，着力推进工业聚集化发展，全面加强园区基础设施建设和市场、服务、金融等软件建设，突出“园中园”引领作用，将保山工贸园区打造成千亿级产业聚集区。落实重点产业发展各项政策措施，全力做强六大产业，加快构建开放型、创新型和高端化、信息化、绿色化现代产业新体系。农特产品加工产业，要以建设万亩规模农业示范区和培育10户农特产品加工企业上市、挂牌为抓手，加快产业基地建设和规模经营主体培育，推动企业升级技术和创建品牌，实现产值444亿元。新材料产业，要加快打造硅系列产品、动力锂离子电池生产、天然气化工、玄武岩纤维、硅藻土基地，实现产值180亿元。轻纺产业，要围绕打造全产业链的目标，加快引进织布、绿色印染、成衣等行业，实现产值200亿元。信息产业，要大力发展数据存储、备份、呼叫中心等产业，实现产值100亿元。旅游文化产业，要以全域旅游和品牌化发展为抓手，加强与凤凰卫视、清华启迪、东方园林等大集团合作，着力把高黎贡山世界自然遗产打造成知名品牌，把腾冲传统村落打造成世界文化遗产，把保山打造成滇西最美生态城市，积极培育养生养老、体育旅游、飞行体验等旅游文化新业态，实现产值559亿元。现代物流产业，要加快专业交易市场、园区产业配套仓储等物流基础设施建设，培育一批现代物流企业，实现产值140亿元。

（四）强力推进生态城市建设，提升城乡人居环境。按照5A级景区标准，以3个万亩生态廊道工程为抓手，着力推进中心城市建设。抓好腾冲国际化山水田园城市、施甸和龙陵山水风光城市、昌宁田园城市建设。加快推进海绵城市建设，20%的建成区达到海绵城市标准。抓紧实施棚户区改造、地下综合管廊、城市轨道交通等重点工程，强化城市与产业园区的功能互补，形成以城聚产、以产促城、产城联动的城镇化发展新格局。着力推进特色小镇建设，加快腾冲市、板桥镇国家新型城镇化综合试点建设，打造板桥镇国家级健康疗养型小镇。依法治理城乡生态环境，强力推进城乡人居环境提升行动，抓实城乡“四治三改一拆一增”和村庄“七改三清”环境综合整治。以“四创两争”为目标，以绿化、美化、彩化、香化为抓手，建设天蓝地绿水清气净的美好家园。

（五）强力推进民生改善，实现发展成果共享。围绕“两不愁、三保障”目标，按照 “五个一批”“五个做成”的要求精准施策、合力攻坚，坚决执行脱贫攻坚“军令状”；强化公共服务和基础设施建设，加快培育特色优势产业，增强贫困地区发展后劲；不断健全贫困县考核、约束、退出机制，确保如期实现脱贫目标，与全国全省同步进入小康社会。健全覆盖城乡的就业创业服务体系，紧扣重点产业开展就业创业培训服务。实施科技重点专项，加强知识产权保护，推进创新型保山建设。巩固提升义务教育，逐步普及高中阶段教育，大力发展学前教育、民办教育等各类教育，推动城乡教育均衡发展。构建现代公共文化服务体系，加大文化遗产保护开发利用力度，促进文化交流，推动文学艺术繁荣发展。健全健康服务体系，推进公立医院综合改革，改善医疗服务质量，加强妇幼保健、疾病防控和健康促进等工作，振兴发展中医药事业。坚持计划生育基本国策，落实好人口发展战略。深入推进民族团结进步示范区建设，加快少数民族和民族地区发展，促进民族团结、宗教和谐。抓好社会保障、社会救助、优抚安置、双拥等工作。统筹推进全民健身、竞技体育和老年教育、社会化养老、妇女儿童等事业发展。切实抓好“七五”普法、地方立法规划、综治维稳、边境管控、安全生产、食品药品安全、防灾减灾等工作，维护社会和谐安宁。

三、2017年重点工作

今年是第四届市人民政府履新之年。我们要坚持稳中求进总基调，以供给侧结构性改革为主线，按照市委四届二次全会提出的发展目标和总体部署，紧紧围绕“六化突破年”“机制完善年”“工业攻坚年”三大主题，突出重点，狠抓落实，促进跨越。

2017年经济社会发展主要指标建议为：生产总值增11%以上；规模以上固定资产投资增25%以上；一般公共预算收入增8%以上；社会消费品零售总额增12%；进出口总额增15%以上；城乡常住居民人均可支配收入分别增10%和12%以上；金融机构存贷款余额分别增15%和13%以上；实际引进市外到位资金增20%以上；居民消费价格涨幅控制在3%以内；单位生产总值能耗下降2.3%，环境质量等指标完成省政府考核任务。

围绕以上目标，我们要努力做好以下八个方面的工作。

（一）全力保持经济较快增长

深化供给侧结构性改革。全面完成省下达的水泥、煤炭等领域去产能任务，严禁新上过剩产能项目；扎实推进咖啡、茶叶、蚕桑等传统产业转型升级，支持企业技术创新、产业链延伸和品牌打造，努力提高产品供给质量效率；有效推动住房回归居住属性，促进农民进城与房地产供给有机衔接，引导市场预期，确保房地产库存保持在合理区间；多措并举稳增长、降成本，认真落实国家、省减税降费政策，用足用好企业用地、用电、物流、社保优惠等政策措施，支持实体经济发展壮大；继续抓好去杠杆、补短板各项工作，有效防风险、惠民生。

切实增加有效投资。强力推进“五网”建设，确保完成投资150亿元以上。加快推进昌保、腾猴、保施和东绕城等6条高速公路建设，力争芒孟高速象达至链子桥段开工，建设农村公路800公里；加大过境铁路建设力度，全面完成保瑞段征地拆迁工作，抓实保山至猴桥、六库铁路前期工作。加快腾冲机场改扩建及航空口岸建设，确保保山机场改扩建和昌宁通用机场开工，腾冲、施甸、龙陵通用机场前期工作取得新进展。加强水源及引调水工程建设，实施好农业综合开发、节水灌溉等项目，加快施甸红谷田等5座中型和腾冲大坡等16座小(一)型水库建设，力争新开工龙陵勐堆等8座中小型水库，完成10万农村人口饮水安全巩固提升任务。加快龙川江流域水电开发，抓好电网规划建设，推进国家天然气利用试验示范区前期工作，争取启动500千伏永昌输变电工程。加大农民安置房建设力度，实施棚户区改造14073套（户），确保完成投资100亿元以上。加强信息化建设，确保信息惠民国家试点城市通过评估验收。

努力扩大消费和进出口。落实促进消费各项政策，大力发展金融、现代物流及信息服务等生产性服务业，积极培育家政、电子信息、文化娱乐、健康养老等新的消费热点。加快推进电子商务进农村综合示范县项目建设。做好“小进规”企业培育，支持更多限额以上企业扩大规模，实现服务业增加值增长11%以上。完善口岸管理服务机制，优化进出口商品结构，加大扶持培育力度，促进进出口总额稳定增长。

（二）全力推进产业转型升级

着力推进农业规模化。突出优质、安全、绿色导向，深入推进农业供给侧结构性改革，促进农民持续增收、农业提质增效。全面加快10个万亩规模农业示范区建设，大力推广公平模式，加大土地流转力度，流转面积达35万亩以上。以示范区企业为龙头，发挥多种形式适度规模经营的引领作用。大力发展“互联网+现代农业”，强化新型经营主体培育，新增省级龙头企业5户、市级龙头企业10户以上，省级农民合作示范社20个以上，打造一批农业庄园、种养大户和家庭农场。落实粮食安全行政首长责任制，稳定粮食生产，确保粮食安全。持续抓好优势产业、特色产业和农特产品加工。确保农业增加值增长5.5%以上。

着力推进工业聚集化。加大“园中园”基础设施建设和土地收储力度，确保年内主干道路全部贯通。强化“园中园”招商引资，推动资金、项目、土地、能源、人才等要素向“园中园”聚集，努力解决“园中园”企业融资、用电、用工等困难问题，确保每个“园中园”新增入园企业亿元以上项目投资额达10亿元以上。加快年产5GW单晶硅棒、富仕通科技、保山复烤厂易地搬迁改造、恒丰纺织44万锭高端纺纱生产线等项目建设进度，争取每个“园中园”建成投产企业2户以上，“园中园”实现产值22亿元以上。全面落实民营经济发展政策措施，民营经济增加值占GDP比重达50.5%以上。加大规模企业培育力度，新增规模以上工业企业31户、小微企业450户，规模以上工业增加值增长13%以上。

着力推进旅游品牌化。抓好全域旅游发展10项重点工作，高标准创建腾冲全域旅游全国示范区。着力推进腾冲国际文旅风情度假区、启迪国际冰雪度假小镇、高黎贡山国家公园、青华海园林生态酒店、昌宁田园城市旅游综合体、施甸怒江大峡谷旅游综合体、龙陵松山大战遗址纪念园等重点项目建设。启动世界温泉公园、世界线性文化遗产和高黎贡山国家农业公园申报工作。启动和顺古镇5A级和北海湿地4A级景区创建，完成潞江坝服务区、松山、银杏村3A级景区创建。拓展航空旅游，新增2条以上省外航线。切实规范和整治旅游市场，提升旅游形象。全年接待海内外旅游者、实现旅游总收入分别增长20%和30%以上。

（三）全力推进城乡一体化发展

着力推进城市生态化。扎实推进中心城市3个万亩生态廊道工程，完成万亩青华海湿地东湖挖掘和绿化、景观、游道工程，争取2018年春节前建成开放；加大万亩生态观光农业园项目招商力度，同步推进土地流转和基础设施、产业基地建设；完成万亩东山生态恢复工程主干道和配套服务设施，建成东山森林公园5个县市区“园中园”。加快推进各县市城市生态廊道建设。大力推进腾冲市、板桥镇国家级新型城镇化综合试点建设，切实抓好2个省级、3个市级新型城镇化试点建设。加快特色小镇建设前期工作。加大城市综合管廊、市政交通、能源通信、电力燃气、污水和垃圾处理等基础设施投资，理顺中心城市供水管理体制，提升供水保障能力。统筹教育、文化、卫生、体育、市场等公共服务配套，积极推进产城融合。

抓实城乡人居环境提升行动。扎实开展城乡“四治三改一拆一增”和村庄“七改三清”环境综合整治行动，着力拆除违法违章、不符合城乡规划、不符合发展标准的建筑，大力整治私搭乱建、占道经营、噪声污染、渣土泼洒、环境卫生脏乱差等问题，打造一批美丽乡村。乡镇生活垃圾处理设施覆盖率达80%，污水处理设施覆盖率达40%；乡镇、建制村公厕覆盖率达100%，60%的村庄生活垃圾得到有效治理。完善城市交通体系，加快停车设施规划建设，开工建设中心城市有轨电车项目。新建、改造城市公厕94座。完成中心城市5个农贸市场的建设改造提升。确保年内隆阳、龙陵、昌宁完成省级生态文明县区创建，中心城市和腾冲卫生城市创建通过国家评审，腾冲创建全国文明城市通过评审，力争昌宁通过国家级园林县城评审、施甸通过省级生态文明县区创建技术评估，全市80%的乡镇完成国家级生态乡镇创建。

（四）全力推进改革开放

深入推进经济体制改革。深化投融资体制改革，切实加强银行、基金、债券和上市融资，健全完善“51+49”“10+3”等合作模式，大力推进政府和社会资本合作，积极推动企业上市、挂牌。推动国资国企改革，加强平台公司建设管理，健全政银企、政保企合作机制，加快县市区担保体系建设，强化对民间投资的激励，增强民间投资意愿。推进电力体制改革，争取年内进入昆明电力交易中心交易，增强电力保障能力。加快沿边金融综合改革试验区建设，积极稳妥发展融资登记服务机构、资本管理公司、互联网金融等新金融组织，切实强化基金筹集和监管。继续深化商事制度改革，健全市场信用体系及监管体系。深入推进财税改革，加大支出优化整合力度，创新扶持方式，盘活存量资金，规范税收与非税收入征管，加强预算和政府债务管理，提高财政收入质量和资金使用效率。

深入推进农村综合改革。推进农村土地所有权、承包权、经营权三权分置改革，基本完成农村土地确权登记颁证工作，开展农村集体资产股份权能改革、土地承包经营权有偿退出和“三权”抵押贷款试点。加快推进农村宅基地使用权、集体建设用地使用权、农村房屋所有权确权登记颁证，建立完善农村产权流转交易市场。扎实推进林业综合改革、农田水利改革，抓好供销和农垦改革。完成第三次全国农业普查。深化户籍制度改革，实行差别化落户政策。加快殡葬改革步伐，积极推行绿色文明殡葬。

深入推进行政体制改革。进一步完善公务用车制度改革，稳步推进公务员分类改革，继续深化事业单位人事制度改革。建立健全责任清单、权力清单、负面清单制度，实现“双随机一公开”监管全覆盖，提高行政效率。

深入推进双向开放。积极服务和融入国家“一带一路”等重大战略，深化与长江经济带、珠三角和环渤海等重点区域的交流合作。加强对缅甸、印度、孟加拉等国开放合作，实现密班公路试验段建成投入使用，推动曼德勒保山缪达工业园和产品展示中心、密支那工业园区规划建设取得新进展，推进友好城市交流。加强腾冲边合区配套建设，基本建成珠宝学院，国际电工电器产业园有2-3户企业建成投产，国家级边境经济合作区申报取得突破。强化园区招商、精准招商、以商招商、产业招商，引进市外到位资金增长20%以上。

（五）全力推进生态文明建设

加强生态建设和环境保护。强化自然保护区、国家公园规范建设与管理，基本完成自然保护区机构改革、国有林场体制改革。严厉打击破坏森林资源违法犯罪行为。着力实施新一轮退耕还林还草、国土绿化、低效林改造、石漠化治理、土壤污染治理修复、农村能源建设工程。完成人工造林10万亩、退耕还林1.5万亩，森林抚育19万亩，低效林改造12万亩。更加注重水生态系统保护，建立河长制，持续抓好东河和各县市城市河流综合治理工程。加大对采石、采矿、采砂、探矿、取土等生态破坏区域的恢复力度，切实保护好森林生态系统。推进水、大气、土壤等污染防治，严格环境执法，改善生态环境质量。

积极推进低碳绿色发展。强化重点企业和行业污染防治，推进节能减排重点项目建设。大力发展绿色经济，实施千家企业节能低碳行动，引导落后产能有序退出，推进集约节约使用土地和水资源。严格建设项目环境准入和节能评估审查，落实污染减排目标责任制，坚决依法处罚、关停排放不达标和违法偷排的企业。

（六）全力保障和改善民生

打好脱贫攻坚战。严把精准识贫和精准脱贫两道关口，确保对象、资金、措施、项目精准。全面推进易地扶贫搬迁，完成省下达搬迁任务。依托龙头企业带动、产业扶持等措施，确保每个贫困村都有产业支撑，增强贫困群众自我发展能力。完成1.9万户农村危房改造、抗震安居工程。抓好教育卫生、水电路等基础设施和集贸市场建设，改善群众生产生活条件。加强项目储备和项目对接，争取浦发扶贫基金186亿元、国开行专项贷款30亿元，加大监管力度，着力实施好基础设施、产业等金融扶贫项目，确保完成省下达减贫目标任务。

健全就业和社会保障体系。实施“贷免扶补”、创业担保贷款、劳动密集型小企业贷款、“两个10万元”微型企业培育工程，多渠道开发就业岗位，以创业带动就业。做好职业技能培训和创业培训，支持园区用工，完成农村劳动力培训18万人，转移5.9万人，新增城镇就业2.3万人，确保高校毕业生就业率达90%以上。深化基本养老保险制度改革，提高城乡居民基本养老金和最低生活保障标准，健全特困人员救助供养制度。

（七）全力保持社会和谐稳定

优先发展教育。切实巩固义务教育均衡发展成果，统筹推动区域城乡校际教育教学资源均衡配置。大力发展学前教育，加强中小学标准化建设，推进“全面改薄”工程和教育信息化“三通两平台”建设应用。全面加快保山教育园区和腾冲职教园区建设，确保9月1日保一中初中部学生宿舍楼投入使用。

强化科技创新。着力推进华大基因现代农业科技示范园项目、清华启迪科技园落地建设。加大研发投入，推进产学研用紧密结合，新建院士专家工作站10个以上，新增国家级高新技术企业20户以上。大连理工大学滇西产业研究院落成。推进保山工贸园区升级为国家级高新技术产业开发区、龙陵工业园区认定为省级高新技术产业开发区，力促保山学院珠宝产业众创空间认定为国家级众创空间。

提升医疗卫生服务水平。继续深化医药卫生体制改革，启动城市公立医院综合改革。加快县综合医院和乡镇卫生院、社区卫生服务机构、村卫生室标准化建设，确保市人民医院完成搬迁。加强公共卫生和疾病预防控制。继续实施关爱妇女儿童健康行动和残疾人精准康复行动计划，提高大众健康水平。

加快发展文化体育事业。深入推进精神文明建设，大力弘扬社会主义核心价值观。加大文化基础设施建设力度，抓紧筹建保山艺术中心。加强文化遗产保护和开发利用。广泛开展“全民阅读”等群众文化活动，积极打造一批文艺精品，提升广播电视服务水平。加强城乡公共体育设施建设，新建和改造一批小区健身设施，持续实施“七彩云南全民健身工程”，推进群众体育与竞技体育协调发展。

强化社会治理。广泛开展“七五”普法宣传教育，推进依法治市。发挥城乡居民自治组织的基础作用，提高治理能力，完善治理体系。推进“6995”信息服务平台升格为“995”，建设集网格化管理、政务服务、医疗服务于一体的便民利民管理服务平台。强化人民调解、行政调解、司法调解的协调联动，落实“三个亲自”，做好信访维稳和矛盾化解工作。抓好食品药品、安全生产监督管理和地质灾害防治。加大民族团结进步示范区创建力度，依法管理宗教事务。深入推进平安保山、法治保山建设，打好禁毒防艾人民战争，为跨越发展提供和谐稳定的社会环境。

（八）全力加强政府自身建设

牢固树立“四个意识”，以建设人民满意政府为目标，以法治作为政府运行的基本准则，以职能转变、能力提升和作风建设为重点，以上率下，严字当头，讲政治、讲团结，敢担当、有作为，求实效、重廉洁，推进依法行政、从严治政、廉洁从政。深入学习贯彻国家、省方针政策和市委决策部署，统筹推进各类教育、培训、学习考察和工作实践，强化担当，落实责任，着力提升履职能力。加强新型智库建设，改进调查研究，健全公示听证、决策咨询、法制审查、专家论证等制度，着力提升决策能力。积极转变政府职能，强化服务意识和机制建设，推进“互联网+政务服务”和公共资源交易标准化建设，开展政务服务中心为民办事“一次办结”试点，着力提升管理服务能力。落实“三种办法”，加大督查问责力度。完善作风建设长效机制，大力整治庸懒散拖，着力提升落实和创新能力。继续推进法治政府建设，积极推进地方立法，严格执行规范性文件备案审查制度，健全政府法律顾问制度，推进行政行为、行政执法规范化，着力提升依法行政能力。自觉接受市人大的法律监督、工作监督和市政协的民主监督，推进政府协商民主，加强与人大代表、政协委员以及民主党派、工商联、无党派人士和人民团体等沟通协商，认真听取意见建议，提高人大代表议案、建议和政协提案办理质量。强化权力监督制约，推进决策、管理、服务、结果公开。健全经济责任审计制度，逐步实现审计监督全覆盖。切实履行全面从严治党“两个责任”，严格落实党风廉政建设“一岗双责”，着力提升拒腐防变能力。

各位代表！风正海阔，自当扬帆破浪；任重道远，更需策马扬鞭！让我们紧密团结在以习近平同志为核心的党中央周围，在省委、省政府和市委的坚强领导下，坚定信心、开拓进取，奋力赶超、率先跨越，用优异的成绩向党的十九大献礼！
